# Validated HPLC-UV Method for Simultaneous Estimation of Paclitaxel and Doxorubicin Employing Ion Pair Chromatography: Application in Formulation Development and Pharmacokinetic Studies

**DOI:** 10.1155/2022/7708235

**Published:** 2022-03-09

**Authors:** Ravi Saklani, Amrendra K. Tiwari, Pavan K. Yadav, Pooja Yadav, Manish K. Chourasia

**Affiliations:** ^1^Division of Pharmaceutics and Pharmacokinetics, CSIR–Central Drug Research Institute, Lucknow 226031, India; ^2^Academy of Scientific and Innovative Research (AcSIR), Ghaziabad 201002, India

## Abstract

Though paclitaxel (PTX) and doxorubicin (DOX) are amongst the most widely used and investigated drug pair for combination chemotherapy but surprisingly, not a single validated HPLC-UV method is available to analyze PTX and DOX simultaneously. So, herein a HPLC-UV method is developed and validated for the same, filling an indispensable gap in the literature. As these two moieties have characteristically different polarities, resolving them under the common chromatographic conditions is a challenging task. Herein, the principle of ion pair chromatography is utilized to resolve these two moieties on a C18 column employing an isocratic mobile phase comprised of acetonitrile and octane sulfonic acid buffer (67 : 37) and detected simultaneously at 231 nm using a UV detector only. The retention time is 4.4 and 7.2 min for PTX and DOX, respectively, with a total analysis time of less than 10 minutes, suitable for the formulation development and research, while LOQ is less than 0.066 *μ*g/ml for both the drugs, suitable for the therapeutic drug monitoring at preclinical and clinical research setup. To substantiate the applicability of the developed method, a nanoformulation coloaded with PTX and DOX was designed and analyzed using the developed protocol. The method is also applied successfully to study the plasma kinetic profile of both the moieties simultaneously in Balb/c mice. Further, the method is validated as per the ICH guidelines fulfilling the unmet need of a validated analytical tool to simultaneously estimate PTX and DOX. Moreover, the results suggest that the principal of common ion chromatography demonstrated here can also be applied further for the simultaneous chromatographic separation of other polar and nonpolar moieties too. Consequently, the reported method surely will advance the toolset required for the precision-based combination chemotherapy.

## 1. Introduction

Paclitaxel (PTX) and doxorubicin (DOX) are the first-line chemotherapeutics that are very frequently used and investigated for combination chemotherapy in different types of cancer management. Furthermore, there is a recent upsurge of interest in development of newer dosage forms for the codelivery of these two moieties for combination chemotherapy [1–8]. Though many validated HPLC methods are easily available for the analysis of PTX and DOX individually, but surprisingly, not a single validated HPLC-UV method is available in the scientific literature for the simultaneous estimation of these two moieties. So, an efficient analytical tool for the simultaneous estimation of PTX and DOX is an unmet need. The most probable reason behind this seems that PTX and DOX have characteristically different polarity and solubility profiles, so resolving them using common chromatographic conditions is a challenging task. Due to this, researchers working on combinational use of PTX and DOX have to analyze them individually employing different dedicated analytical techniques for each drug [3, 6, 9–15].

For instance, in a pharmacokinetic study conducted on breast cancer patients by the group of Moreira, they employed two different HPLC methodologies for the estimation of PTX and DOX, respectively. Owing to the difference in their polarities, DOX was estimated using normal-phase chromatographic conditions and PTX using reverse-phase (RP) HPLC [6]. Similarly, pharmacokinetic study reported by Gianni et al. employed two different HPLC methods for the estimation of PTX and DOX, respectively [14]. Analytical methods reported by Ahmed et al. and Markovsky et al. employed fluorescence spectroscopy for the analysis of DOX and HPLC separately for the analysis of PTX [10, 15]. Similarly, methods reported by Wang et al., Duong et al., Lv et al., Liu et al., and Wang et al. used UV-visible spectroscopy for estimation of DOX and HPLC method separately for analysis of PTX [3, 11–13, 16].

Only report where an attempt has been made to analyze PTX and DOX simultaneously along with 3 more anticancer agents is by Larson et al. [17]. The method reported has very high runtime of over an hour with PTX detected after 39 minutes of runtime. The coefficient of variation was as high as up to 19.7% and 15.5% for PTX and DOX, respectively. Moreover, the linearity range is also very narrow and detection limits for PTX and DOX are not sufficiently sensitive rendering method unsuitable for analytical purpose in formulation development and bioanalytical studies. Other than these, due to unavailability of a validated HPLC method, LC-MS is another analytical tool that is frequently employed for the coestimation of PTX and DOX [1, 17]. LC-MS is a sensitive analytical tool but at the same time very expensive and not readily available compared to HPLC.

In the light of all these facts, our work is aimed at providing an alternative analytical tool for coestimation of PTX and DOX, more efficient than any other method reported earlier. To the best of our knowledge, there is no validated HPLC-UV method reported yet for the estimation of PTX and DOX simultaneously. So, in this report, a validated HPLC method is reported for the simultaneous estimation of PTX and DOX. A validated method that can be easily executed using a readily available UV detector tagged HPLC system that is exponentially cheaper than conventionally employed fluorescence or mass spectroscopic detector accompanying chromatography instruments. The method is designed to be suitable for application in new dosage form development and therapeutic drug monitoring of PTX and DOX. The PTX- and DOX-coloaded liposomes are developed and analyzed in this work to substantiate the protocol's applicability. The application of the developed method is further applied to study the plasma pharmacokinetic of the PTX and DOX coadministered to Balb/c mice via developed nanoformulation.

## 2. Materials and Methods

### 2.1. Materials

PTX and DOX were received as a generous gift sample from Alembic Pharmaceuticals, Vadodara, India. 1-Octane sulfonic acid sodium salt (OSA), orthophosphoric acid (OPA), phosphatidyl choline (egg lecithin), and cholesterol were procured from Sigma, USA. Acetonitrile (ACN) of HPLC grade was procured from Merck, India. Milli-Q water purification system (Millipore, USA) was used for filtered triple distilled water.

### 2.2. Method

#### 2.2.1. RP-HPLC Instrumentation and Chromatographic Conditions

A Shimadzu HPLC system integrated with LC 10 ATVP pump and a Rheodyne® injector (model 7125, 20 *μ*l loop) was used for chromatographic method development and validation. Chromatographic resolution was accomplished on a RP LiChrospher® C18 column (100, 250 mm × 4.6 mm, 5 *μ*m; Merck), at 35°C of column temperature. Both the moieties eluted using an isocratic mobile phase comprised of aqueous buffer (0.025% *w*/*v* OSA; pH adjusted to 3.0 with OPA) and ACN mixed in a ratio of 37 : 63 parts, respectively. The total flow rate of mobile phase was 1 ml/min, with a total runtime of 10 minutes. The eluent was monitored at 231 nm for both the drugs using a Shimadzu SPD-M10 UV-PDA detector.

#### 2.2.2. Preparation of the Stock Solutions

Appropriately weighed amount of PTX and DOX was dissolved in ACN and water, respectively, to make a primary stock solution of 1 mg/ml concentration of both the drugs. A secondary stock solution of 100 *μ*g/ml (each drug) was prepared further by mixing and diluting primary stock solutions in a mixture of acetonitrile and water (1 : 1). The stock solutions were stored at −20°C until further use. Working dilutions were made from the secondary stock solution each time before analysis using mobile phase.

### 2.3. Method Validation

The assay method for PTX and DOX was validated for system suitability, linearity, accuracy, precision, robustness, stability, and specificity as per previous report of Singh et al. and in accordance with the ICH guidelines [18, 19]. The value of relative standard deviation (%RSD) was calculated for different analytical parameters, and validity was assessed on the basis of %RSD value.

### 2.4. Applicability of the Method

#### 2.4.1. Preparation and Analysis of PTX and DOX Coloaded Nanoformulation

PTX- and DOX-loaded liposomes were prepared and evaluated for specificity, entrapment, and loading efficiency to evaluate the applicability of the developed method. The multilamellar liposomal vesicles were formulated employing standard solvent evaporation and hydration method [20]. Briefly, phosphatidylcholine, cholesterol (7 : 3 mole), and PTX were dissolved in chloroform to form a clear solution of oil phase. The organic solvent from the lipid-phase mixture was evaporated under vacuum using rotary evaporator to form a dried thin lipid film. The dried lipid film was subsequently hydrated with the normal saline solution of DOX by vigorous vortexing. The resultant hydrated liposomal vesicles were collected by centrifugation at 14000 *g* for 15 min and followed by washing with PBS solution [13]. Blank nanoformulation was prepared using the same procedure but without adding the drugs. The developed formulation was lyophilized and further analyzed for specificity, matrix effect, entrapment, and loading efficiency utilizing the developed HPLC method [21]. To evaluate the matrix effect and specificity of the developed method, blank formulation was fortified with known amount of drug and dissolved in methanol to disrupt the nanocarrier [22]. The samples were then analyzed by the developed HPLC method for PTX and DOX in the presence of formulation components. For loading and entrapment efficiency calculation, the drug-loaded formulation was weighed and processed similarly as in specificity assay. The total loaded drug in the formulation was estimated and expressed as percentage fraction of the total formulation weight (loading efficiency) and weight of the total drug added to the formulation (entrapment efficiency) [23, 24].

#### 2.4.2. Bioanalytical Study

The specificity of the developed analytical method was determined in plasma samples. The 100 microlitres of plasma sample was spiked with known concentrations of both the drugs and incubated with drug for 0.5 h. This was followed by addition of precipitant and extracting solvent (methanol and ethyl acetate (4 : 1)) and vortex for 15 minutes. The sample was centrifuged, and the supernatant was collected. The supernatant sample was evaporated under vacuum, and final volume was reconstituted with the mobile phase. The reconstituted sample was injected into HPLC column and analyzed. All the samples were analyzed in triplicate, and interfering peaks were observed visually at the retention times (Rt) for PTX and DOX.

#### 2.4.3. Pharmacokinetics Study

The applicability of the developed HPLC method was further assessed to study the plasma kinetics of the PTX and DOX coadministered to mice via developed formulation. The *in vivo* plasma kinetic was studied in 8-10-week-old female Balb/c mice (20-25g), in accordance with the approved guidelines of Institutional Animal Ethics Committee of CDRI, India. The developed formulation was administrated intravenously to mice at a dose level equivalent to 10 and 6 mg/kg of PTX and DOX, respectively. At predetermined time intervals, blood sample was collected and centrifuged at 3000 *g* to separate the plasma. Plasma samples were processed and analyzed as mentioned in the bioanalytical specificity assay. Different pharmacokinetic parameters were calculated employing Microsoft Excel add-in program PK Solver, employing the noncompartmental analysis [25].

## 3. Results and Discussion

### 3.1. Method Development

**Figure 1:**
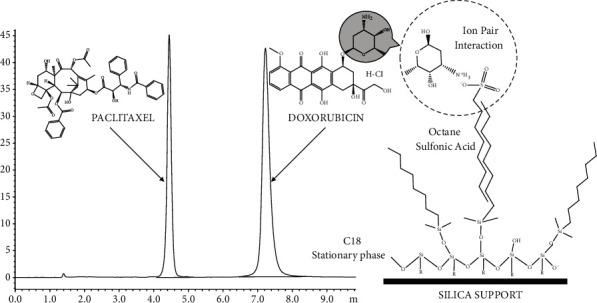
Ion pair chromatography employed for resolution of PTX and DOX; representative chromatograms of PTX (4.4 min) and DOX (7.2 min) detected at 231 nm.

If two moieties with contrasting polarities and solubility profile have to be resolved and analyzed simultaneously in a reasonable timeframe, the demands on both the mobile and stationary phase are high. Paclitaxel is a highly hydrophobic nonpolar moiety that requires reverse-phase HPLC conditions. But when need to resolve with DOX which is a hydrophilic and polar moiety, it is a bit tricky. DOX being a polar moiety needs a special treatment to retain in a RP C18 stationary column. In our previous report, we employed the principle of ion pair chromatography to retain bendamustine hydrochloride (a polar moiety) satisfactorily on a nonpolar C18 stationary phase [18]. It appeared as a plausible approach to try the same principle with DOX, another polar moiety. So, herein too, we attempted principle of ion pair chromatography employing OSA, to bring PTX and DOX on the same platform. OSA was added to the mobile phase to increase the affinity of the DOX for the stationary phase. The negatively charged polar head of OSA formed the ion pair with the positively charged amino group of the DOX. On the other hand, the nonpolar alkyl chain increased the affinity of a newly formed ion pair to the C18 stationary phase (Figure 1). This inversed the elution affinity of both the drugs with PTX eluted at 4.4 minutes well before DOX but well after the solvent front while DOX eluted with good resolution after PTX at 7.2 minutes (Figure 1). Otherwise, the absence of an ion pair reagent retention and resolution of DOX on RP column was inadequate. It was eluted in the dead volume or faced high interference by the solvent front. Addition of OSA to the mobile phase resolved the issue of DOX retention on a C18 column.

**Figure 2:**
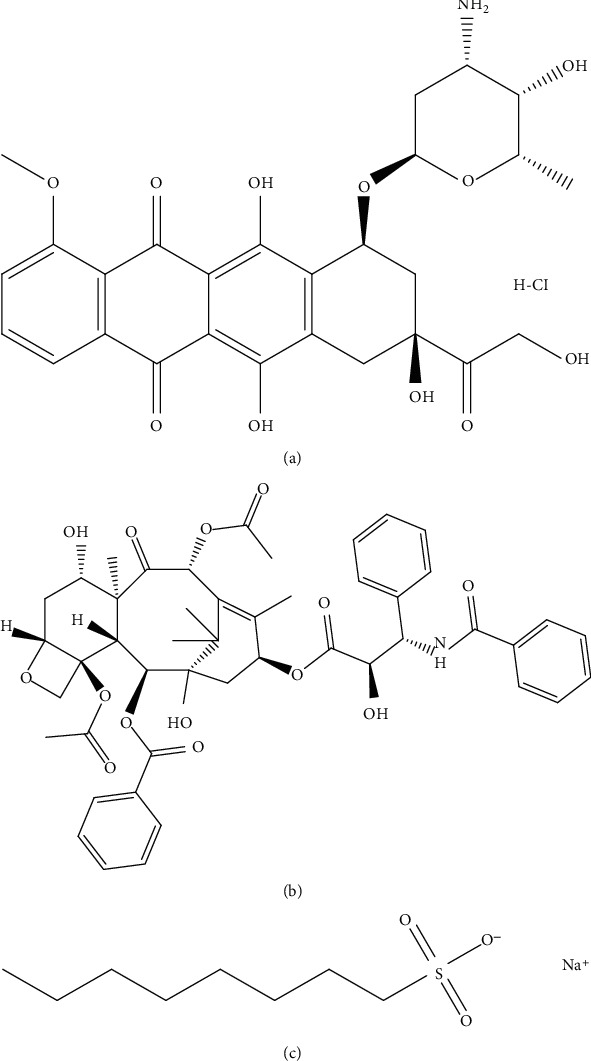
Chemical structure of (a) doxorubicin hydrochloride, (b) paclitaxel, and (c) octane sulfonic acid.

Furthermore, DOX is an amphoteric drug molecule comprised of protonable amino group in its glycon moiety and deprotonable phenolic groups in its aglycone moiety (Figure 2) [26]. Thus, it exhibits a continuous variable charge between negatively charged, neutral, and positively charged species and exists as a zwitterion, deterring any coherent interaction with the stationary phase. This caused distorted peak with very high tailing (tailing factor > 2.4) and inconsistent retention time. So, to tackle this erratic behaviour of DOX, chromatographic elution of DOX was attempted by adjusting pH of the aqueous component in mobile phase to 3 using OPA. The acidic pH imparted predominantly a positive charge to the DOX and it existed in the form of protonated NH_3_^+^ ion leading to a consistent interaction with the negatively charged sulphate ion in OSA. These conditions provided sharp peak of DOX with constant Rt. On the other hand, PTX which is a predominantly nonpolar and hydrophobic moiety remained unaffected by these ionic and electrostatic manipulations, and elution of PTX could be easily controlled by simply altering the ratio of aqueous and organic content in the mobile phase. This dual mechanism provided a great degree of control over elution of both the moieties simultaneously. Thus, mobile phase composed of 0.025% *w*/*v* aqueous solution of 1-octane sulfonic acid (pH adjusted to 3 by OPA) and ACN (37 : 63) was finalized with C18 column as a stationary phase. The resulted chromatographic conditions provided satisfactory resolution of DOX at a retention time of 7.2 min and PTX at 4.4 min, with sharp peaks, and good resolution with respect to each other and solvent front in a reasonable timeframe suitable for formulation development and therapeutic drug monitoring application.

### 3.2. Method Validation

The assay method for PTX and DOX was validated for system suitability, linearity, accuracy, precision, robustness, stability, and specificity as per ICH guidelines. The % RSD values of the analytical parameters less than 2% are considered under accepted limits.

#### 3.2.1. System Suitability

System suitability was assessed on the basis of 6 replicate injections of standard which revealed that analytical parameters are in acceptable range with % RSD value always less than 2. The retention time for PTX and DOX is 4.4 and 7.2 min, respectively, showing good resolution with respect to each other and the solvent front. The % RSD value of the retention time was less than 0.5 for both of the drugs, demonstrating good reproducibility on repeated injections. The value of tailing factor for both PTX and DOX peaks was inside a range of 0.91 to 1.25 in all the peaks showing fine peak symmetry. The theoretical plate number was always more than 2000 indicating good column efficiency throughout all the chromatographic runs.

#### 3.2.2. Linearity

**Figure 3:**
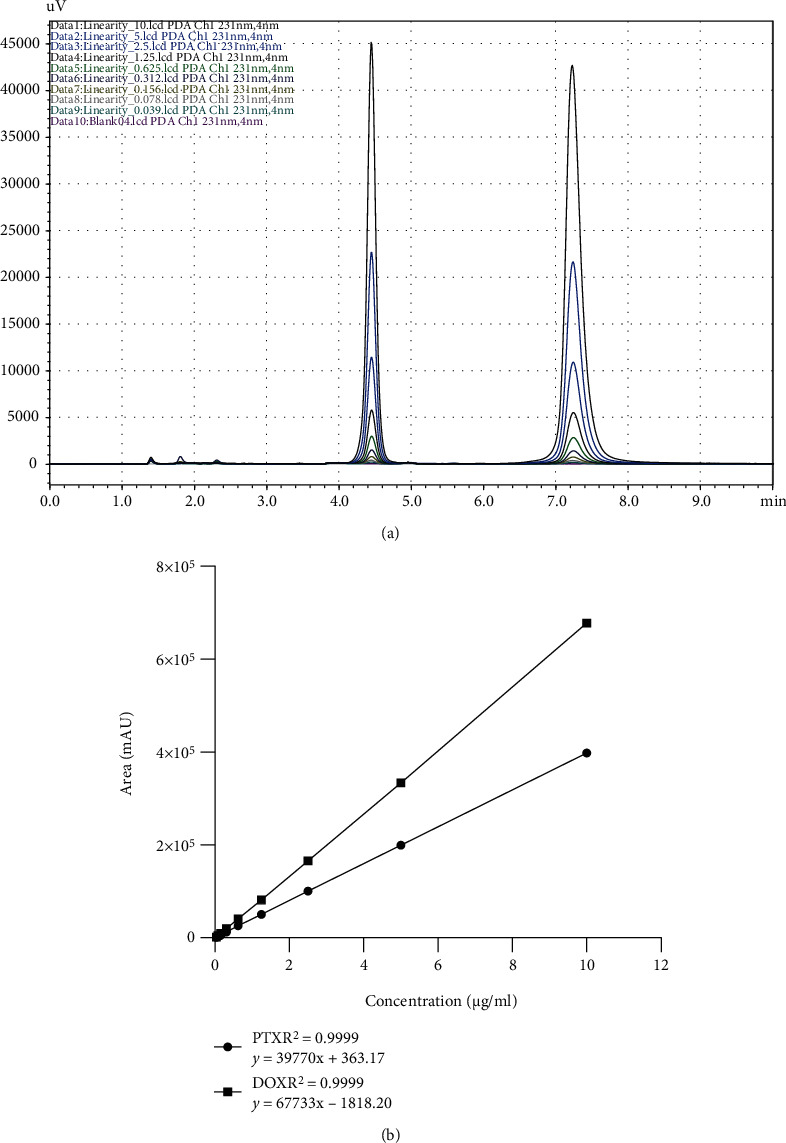
Linearity of PTX and DOX. (a) Overlay chromatogram of different concentration injections. (b) Calibration plot between concentrations (*μ*g/ml) and peak area (mAU) of PTX and DOX with regression line equation *y* = *mx* + *c*, where *x* is the concentration (*μ*g/ml), *y* is the peak area (mAU), *m* is the slope, and *c* is the intercept on the *y*-axis.

The standard calibration curve was plotted between a concentration range of 0.039 to 10 *μ*g/ml for both the drugs to analyze the formulation and bioanalytical samples (Figure 3). The limit of detection (LOD) and limit of quantification (LOQ) values for both drugs were calculated mathematically using residual standard deviation (*σ*) and the slope average (*S*) of the calibration curve as per ICH guidelines (LOD = 3.3 *σ*/*S*; LOQ = 10 *σ*/*S*) [19]. The calibration curve was linear with value of correlation coefficient (*r*^2^) more than 0.99 for both analytes, calculated from the calibration line [27]. Different parameters of regression curve are shown in Table 1.

#### 3.2.3. Accuracy and Precision

The results for accuracy and precision are expressed in Table 2. The variations were measured in terms of % RSD found to be less than 2% for the both drugs, indicating that the developed method is accurate with % recovery for both drugs which is between 99 and 101% in analytical samples, while the plasma recovery (Table 2) was found to be more than 85% and RSD less than 5% for both of the drugs at different concentration levels. The precision results (Table 2) demonstrate the familiarity of the result of different sample injections acquired and establish that the developed method is precise.

#### 3.2.4. Standard Solution Stability

As shown in Table 3, sample solutions of PTX and DOX were found to be stable up to 24 hours in both refrigerated and room temperature conditions. Negligible degradation of DOX was observed at refrigerated condition after 48 hours and after 24 hours at room temperature. PTX solution was stable in refrigerated condition for over 72 hours of the observation period. Negligible degradation was observed at room temperature condition after 72 hours.

#### 3.2.5. Method Robustness

Table 4 shows the % recoveries for both drugs were between 97 and 103% compared to the area of sample injections analyzed by a standard finalized protocol, validating robustness of the developed method (Table 4). However, there were slight changes in the retention times on alterations of the mobile-phase composition and the flow rate.

#### 3.2.6. Specificity of Analysis in the Presence of Formulation and Plasma Samples

**Figure 4:**
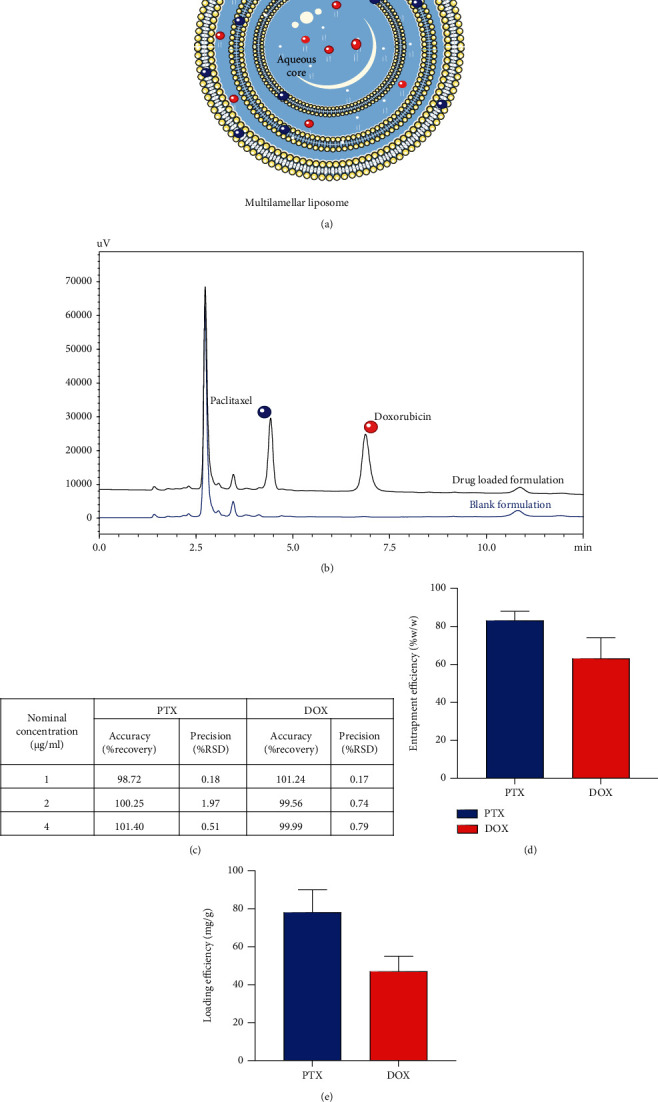
Application of the developed method in evaluation of the combination formulation. (a) Multilamellar liposomal formulation coloaded with the PTX and DOX in lipid and aqueous compartment, respectively. (b) Chromatogram depicting specificity of the analytes with respect to the formulation components. (c) Matrix effect of the formulation components on analysis of PTX and DOX, (d) entrapment efficiency, and (e) loading efficiency of the developed formulation analyzed by the developed HPLC method.

**Figure 5:**
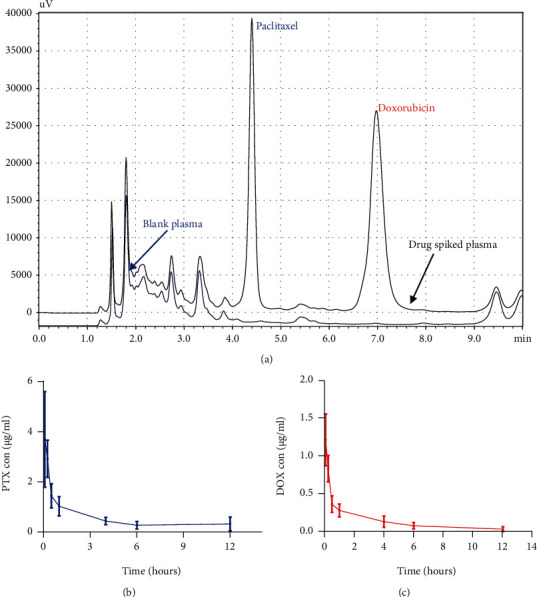
Application of the developed method to study the plasma kinetic of PTX and DOX in Balb/c mice. (a) Chromatogram depicting specificity of the analyte with respect to plasma proteins. (b) PTX and (c) DOX plasma concentration and time profile following intravenous administration of liposomal formulation loaded with PTX and DOX. Values are expressed as the mean ± SD; *n* = 3.

As shown in Figures 4(b) and 5(a), the result confirms no intrusive peaks found at the retention times for PTX and DOX in the formulation and plasma samples. This implies that the method is specific. The plasma recovery (Table 2) was found to be more than 85% for both of the drugs at different concentration levels suitable for bioanalytical analysis. Furthermore, the specificity and matrix effect of the formulation component were evaluated at three dose levels (1, 2, and 4 *μ*g/ml), employing a standard addition method [22]. The developed method is found to be specific for the coestimation of the PTX and DOX in the developed formulation system with matrix effect ≤ ±2% (Figure 4(c)).

### 3.3. Applicability of the Method

#### 3.3.1. Analysis of the Developed Nanoformulation

To illustrate the applicability of the method in determination of percent assays, a nanoformulation coloaded with PTX and DOX was prepared (Figure 4(a)). A phospholipid-based multilamellar liposomal formulation was designed, owing to the amphiphilic nature of the developed formulation that accommodated PTX and DOX in its lipid bilayers and aqueous compartment, respectively. The entrapment and loading efficiency of the developed formulation was analyzed by the developed method (Figures 4(d) and 4(e)). The better loading efficiency of the PTX (78 ± 12 mg/g of formulation) was expected compared to DOX owing to lipophilic nature of the PTX, which favoured its interaction with the lipidic bilayer. On the other hand, DOX (47 ± 8 mg/g of formulation) owing to its hydrophilicity tends to leak out from the liposomal assembly to external aqueous media. The developed method is found to be specific for the coestimation of the PTX and DOX in the developed formulation system without any significant interference from the formulation components.

#### 3.3.2. In Vivo Pharmacokinetic Studies

The developed method was further employed to study the plasma kinetic in Balb/c mice following intravenous administration of the developed formulation loaded with PTX and DOX. The plasma concentration vs. time profiles of PTX and DOX is shown in Figure 5, and pharmacokinetic parameters analyzed with noncompartmental analysis are shown in Table 5. The mentioned method is found to be suitable for the simultaneous estimation of the PTX and DOX in the plasma samples with a total plasma recovery of more than 85% at different dose levels.

## 4. Conclusion

A simple and cost-effective HPLC-UV method for the coestimation of PTX and DOX is developed and validated, as per ICH guidelines. The mentioned method shows good selectivity, reproducibility, and accuracy, with an analysis time of less than 10 min, using RP HPLC conditions and a UV detector only despite of extremely different physiochemical properties of the analyzed moieties. Further validation analysis of the developed method showed that PTX and DOX can be efficiently qualified and quantified for the development of new dosage forms and bioanalytical studies employing the method mentioned. This enabled the estimation of loading and encapsulation efficiency of PTX and DOX in the developed nanoformulation as well as measurement of both drugs in bioanalytical plasma samples for pharmacokinetic study. The developed HPLC method fulfils the unmet need of an efficient analytical tool that can quantify the PTX and DOX simultaneously. It enables the development and evaluation of new dosage forms and therapeutic drug monitoring for combination chemotherapy with PTX and DOX. It surely will advance the toolset required for the precision-based combination chemotherapy.

## Figures and Tables

**Table 1:** Regression curve parameters for PTX and DOX.

Parameters	PTX	DOX
Range of calibration curve (*μ*g/ml)	0.039-10	0.039-10
Detection wavelength (nm)	231	231
Retention time (minute)	4.4	7.2
Correlation coefficient (*r*^2^)	0.9999	0.9999
Slope	39770	67733
Intercept	363.17	-1818.20
LOQ (*μ*g/ml)	0.066	0.038
LOD (*μ*g/ml)	0.021	0.012

**Table 2:** Accuracy and precision of the method.

Nominal concentration (*μ*g/ml)	Intraday		Interday		Plasma	
	Accuracy (% recovery)	Precision (% RSD)	Accuracy (% recovery)	Precision (% RSD)	Recovery (%)	% RSD
PTX						
0.5	99.27	0.89	99.51	0.947	87.74	4.79
1	100.74	0.24	100.46	1.428	85.41	1.34
1.5	99.88	1.07	99.43	1.088	93.96	1.06
DOX						
0.5	100.20	0.12	100.03	1.494	89.11	0.58
1	100.93	0.65	100.40	1.081	90.88	2.73
1.5	99.98	0.13	100.46	1.232	86.82	0.73

**Table 3:** Stability of PTX and DOX samples expressed as percentage of their initial concentration on storage for 3 days under refrigerated and room temperature condition.

Analyte	Concentration	Time (hrs)	Recovery (%)	% RSD
Intraday (5°C)				
PTX	1.0	0	101.06	0.04
		12	101.08	0.07
DOX	1.0	0	100.21	0.76
		12	99.46	1.30
Interday (5°C)				
PTX	1.0	24	100.21	0.55
		48	100.09	0.64
		72	99.03	1.38
DOX	1.0	24	98.83	1.75
		48	98.45	2.02
		72	96.06	3.71
Intraday (25°C)				
PTX	1.0	0	101.06	0.04
		12	101.41	0.30
DOX	1.0	0	100.21	0.76
		12	99.15	1.50
Interday (25°C)				
PTX	1.0	24	99.52	1.04
		48	99.03	1.38
		72	98.12	2.02
DOX	1.0	24	96.97	3.05
		48	96.14	3.64
		72	96.06	3.71

**Table 4:** Robustness expressed in percent mean recovery and percent relative standard deviation for PTX and DOX.

Parameter	Rt	Recovery (%)	%RSD
PTX			
Mobile-phase ACN : water (68 : 32)	3.67	99.82	0.138
Mobile-phase ACN : water (63 : 37)	4.41	98.20	1.318
Mobile-phase ACN : water (58 : 42)	5.60	98.77	0.906
Flow rate (0.9 ml/min)	4.86	102.46	1.804
Flow rate (1.0 ml/min)	4.38	97.77	1.638
Flow rate (1.1 ml/min)	3.99	99.44	0.413
Column temp. (40°C)	4.36	98.30	1.256
Column temp. (35°C)	4.39	97.50	1.818
Column temp. (30°C)	4.40	99.03	0.679
DOX			
Mobile phase (68 : 32)	7.53	100.64	0.451
Mobile phase (63 : 37)	7.46	101.05	0.744
Mobile phase (58 : 42)	7.28	99.59	0.297
Flow rate (0.9 ml/min)	7.74	100.34	0.240
Flow rate (1.0 ml/min)	7.48	100.68	0.485
Flow rate (1.1 ml/min)	6.35	99.07	0.660
Column temp. (40°C)	6.95	100.98	0.698
Column temp. (35°C)	7.34	100.85	0.603
Column temp. (30°C)	7.39	101.64	1.154

**Table 5:** Plasma pharmacokinetic parameters of PTX and DOX following intravenous administration of liposomal formulation loaded with PTX and DOX in Balb/c mice.

Pharmacokinetic parameters	PTX (10 mg/kg)	DOX (6 mg/kg)
*C*_max_ (*μ*g/ml)	3.67	1.20
*t*_1/2_ (h)	3.57	3.45
Clearance (ml/h/kg)	1.20	3.23
AUC_0__-∞_ (h/*μ*g/ml)	8.29	1.85
Mean residence time (h)	6.33	3.89

## Data Availability

The analytical data used to support the findings of this study are included within the article. Furthermore, the raw data if required is available from the corresponding author upon reasonable request.
